# Examination of Pediatric Burn Incidence and the Impact of Social Determinants of Health in Florida

**DOI:** 10.7759/cureus.57035

**Published:** 2024-03-27

**Authors:** Devon Durham, Christopher Rennie, Kelsey Reindel

**Affiliations:** 1 Medicine, Nova Southeastern University Dr. Kiran C. Patel College of Osteopathic Medicine, Clearwater, USA; 2 Osteopathic Principles and Practice, Nova Southeastern University Dr. Kiran C. Patel College of Osteopathic Medicine, Clearwater, USA

**Keywords:** emergency department, pediatric trauma, burn injury, public health and safety, social determinant of health

## Abstract

Introduction

Burn injuries are a major mechanism of trauma worldwide, caused by friction, cold, heat, radiation, chemical, or electric sources. Most often, burn injuries occur due to heat contact from hot liquids, solids, or fire, termed scald burns and flame burns, respectively. These types of injuries are complex and carry major injury and mortality risks, especially in pediatric populations. Burn trauma prevention has been a major focus in the US, with initiatives to increase public health outreach and safety measures. Unfortunately, children in socioeconomically disadvantaged situations may face these types of injuries at disproportionately higher rates, and we aim to highlight these disparities, if any, within our Florida community.

Materials and methods

This study was designed as a retrospective observational analysis using publicly available data from the Florida Health Community Health Assessment Resource Tool Set (CHARTS). Data was extracted for nonfatal burn injuries resulting in ED visits in the years 2018-2020. This data was limited to those ranging from 0 to 19 years old and converted to rates of burn injuries per 100,000. Sociodemographic details for each county were recorded from County Health Rankings & Roadmaps and compared with burn data in each respective county. Frequencies were generated for categorical data, and statistical analyses for burn rates and sociodemographic details were performed with a generalized linear model using a Poisson distribution and bivariate correlation for a p < 0.05.

Results

In Florida, the median annual burn rate per 100,000 was 136 (IQR: 96-179), with Jackson county holding the highest rate of 323 and Glades, Hardee, and Lafayette each holding a rate of 0. Of the 18 socioeconomic factors examined, a total of five were found to have no statistically significant effect on nonfatal burn injury ED visits: severe housing problems, percentage of Asians, teen births, percentage of children (<18 years) in poverty, and severe housing cost burden. The two most important factors to be found in nonfatal burn ED visits of pediatric patients were the percentage of those younger than 19 years old without health insurance and the average grade level performance of third-grader reading scores. When adjusting for the small sample size using Firth’s bias-adjusted estimates and overdispersion, both reading scores and those without insurance play a significant role in pediatric burn injuries. For each increase in a single point in reading scores, the incidence rate ratio decreases by 97.1% (95% CI). For every percentage increase in children insured, there is a 28.8% decrease in pediatric burn injuries (95% CI).

Conclusions

This analysis highlights increased pediatric burn rates across multiple social determinants of health (SDOH) in all 67 Florida counties. The findings here demonstrate that there may continue to be a disproportionate distribution of burn rates among lower and higher sociodemographic areas. This study further highlights this trend within the Florida community, and continued research will be necessary to meet the needs of lower sociodemographic areas to improve burn rates in vulnerable populations, such as children, who are at increased risk of injury.

## Introduction

Burn injuries are a major mechanism of trauma worldwide, with thousands of individuals seeking treatment each year [1]. These injuries are often caused by friction, cold, heat, radiation, chemical, or electric sources. Moreover, most burn injuries are caused by heat from hot liquids, solids, or fires, termed scald burns and flame burns, respectively [1]. Burn injuries can cause major tissue destruction and are determined by size and depth, but they also involve multisystem dysfunction that often leads to further injury [1,2]. For example, the initial burn resuscitation phase is the hypodynamic, or “ebb phase,” which lasts for approximately 24-72 hours and is characterized by increased vascular permeability, fluid shifts, and edema that result in intravascular volume depletion [3,4]. Furthermore, direct vascular injury from burns causes the release of inflammatory mediators that lead to leakage of plasma proteins into the interstitial space, resulting in decreased capillary oncotic pressure [3-5]. In this way, the initial trauma from a burn causes immediate damage as well as a chain reaction of events, further increasing injury and mortality.

In the clinical setting, and especially with children, it is important to estimate burn severity via total body surface area (TBSA). The subdivision of burns is classified by the percentage amount of TBSA and by increasing depth: superficial, superficial partial thickness, deep partial thickness, and full thickness [4]. These factors clue clinicians toward severity and potential complications and trigger a series of treatment algorithms. Beyond the systemic and local reactions to thermal injury, there is additional damage that can add to morbidity and mortality. Inhalation injuries represent a major complication that can be anticipated via prudent TBSA assessment and clinical exam. These injuries compromise the airway and can lead to significant airway swelling, atelectasis, infection, and even systemic toxicity, such as exposure to carbon monoxide [6]. Inhalation injuries alone account for 5,000-10,000 deaths per year in the US [7].

It is evident that burn injuries lead to the development of multisystem complications, but they also lead to devastating impairments of emotional well-being and quality of life [8]. These injuries can require long-term treatment, multiple reconstructive surgical procedures, and long hospital stays that increase the cost of health care and are often accompanied by additional socioeconomic burdens for burn victims and their families [8]. It is not surprising that burn injuries are more common in populations with lower socioeconomic status (SES) and delayed developmental growth, such as areas with a lack of basic safety education [8-10]. At-risk populations tend to face challenges in safety resources, housing instability, access to healthcare, and medical comprehension, all culminating in both increased opportunity for burn injury and increased severity.

Although the US has significantly lower burn injury rates than other areas of the world, there remains a disparity within the US, with a disproportionately higher incidence affecting regions such as Alaska and southeastern states [10,11]. The location represents a major social determinant of health (SDOH) that, in many ways, can be uncontrollable for certain populations. This finding is important to highlight, as there is a gap in the literature surrounding the potential geographic variances that may be causal.

Additionally, vulnerable populations, such as children, have been shown to have increased morbidity and mortality rates associated with burns [10,12]. Children have been found to have increased potential for significant psychosocial (i.e., anxiety, depression, and post-traumatic stress disorder) and physical (i.e., decreased mobility and pain) sequelae [12]. Although educational programs, the introduction of smoke alarms and detectors, and controlled hot water in households have decreased burn incidence rates and severity, there are additional gaps in access to these programs [13]. This study aims to add to the limited body of literature regarding these potential socioeconomic factors and highlight areas of disparity, specifically within the Florida community.

This article was previously presented as a poster and meeting abstract at the Dr. Kiran C. Patel College of Osteopathic Medicine (KPCOM) Office of Graduate Medical Education Student/Intern/Resident/Fellow Annual Research Poster Competition on April 7, 2022.

## Materials and methods

Study design and patient population

This study was performed as a retrospective observational analysis using the Florida Health Community Health Assessment Resource Tool Set (CHARTS), which is a statistically valid public health monitoring database. Data was extracted regarding the number of nonfatal ED visits for the state of Florida and all 67 counties for the ages of 0-19 years old. For the purposes of this study, 19 years old was considered a pediatric age, as supported by the American Academy of Pediatrics. The mean was taken from 2018-2020 and converted to rates of burn injuries per 100,000. Next, using County Health Rankings & Roadmaps, we collected demographic variables for each county along with various socioeconomic factors. The independent variables representing the SES of each county were collected from 2018-2020, and the mean was calculated to match burn rate data.

Variables of interest

Sociodemographic data from County Health Rankings & Roadmaps included the total pediatric population, percentage of poor or fair health, teen birth rate, percentage of uninsured, percentage of uninsured children, primary care provider (PCP) rate, mental health provider rate, high school graduation rate, percentage of single-parent households, percentage of severe housing problems, child mortality rate, percentage of disconnected youth, percentage of children eligible for free or reduced lunch, and race or ethnicity rates.

Burn rates provided by Florida Health CHARTS were collected by mechanism of injury: fire, flame, and hot object or substance. It is important to note that the data extracted did not distinguish burns as a result of abuse, and thus this was not analyzed. Only data in the following age ranges of less than one year, one to four years, five to nine years, 10 to 14 years, and 15 to 19 years old were collected. Total counts of less than five were reported as such, and exact numbers could not be extrapolated, presenting a statistical limitation to this study. In these instances, the counts were listed as zero for the purposes of this analysis.

For a few variables of interest, some counties did not have data reported. For the counties that lacked data for the aforementioned sociodemographic variables of interest, we excluded them for the purposes of analysis. Aside from data availability, injury mechanism, and age, no additional exclusion criteria were utilized.

Statistical analysis

Descriptive statistics were generated, including percentages for discrete data variables and medians and the IQR for continuous variables. We ran a generalized linear model using a Poisson distribution for our dependent variable of pediatric burn rates against our various sociodemographic factors to include all factors in addition to a bivariate correlation for individual variables against burn rates. The linear regression F test was used to analyze the linear relationships among variables. The level of significance was set at 0.05. All statistical analyses were performed using JMP Statistical Software version 16.2.0 (SAS Institute Inc., Cary, North Carolina, US).

## Results

**Figure 1:**
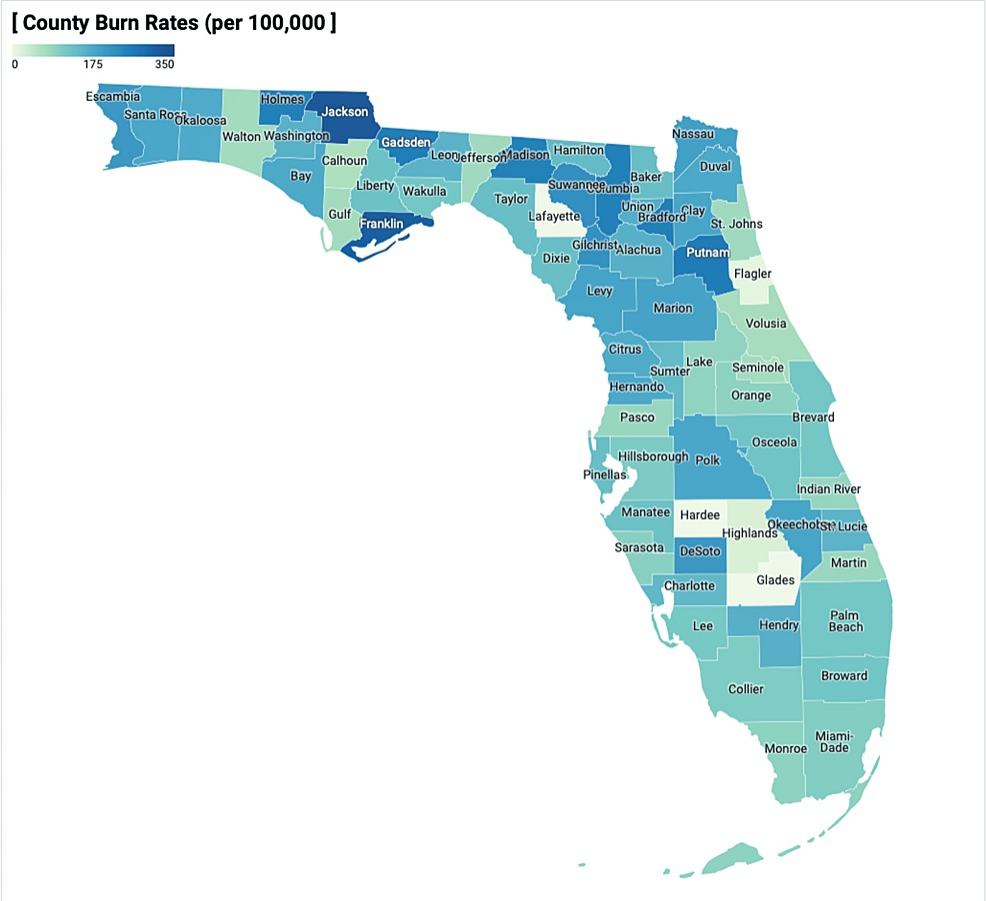
Pediatric burn rates by Florida county Image credit: Devon Durham

The median annual burn rate for pediatric patients in each county was 136 per 100,000 (IQR: 96-179). The county with the largest annual burn rate was Jackson, with a mean annual pediatric burn rate of 323 per 100,000 children. The counties with the lowest pediatric burn rates were Glades, Hardee, and Lafayette, which were reported as zero since counts of less than five were not reported by Florida Health CHARTS. Pediatric burn rates for each county are shown in Figure 1 below.

Table 1 outlines each variable used to determine possible factors that play a role in pediatric burn injuries for all counties. It is important to note that data is represented in percentages as reported by the County Health Rankings & Roadmaps service. Of note, the PCP rate was reported as unavailable for Liberty county. Upon further investigation from the 2013 Liberty County Community Health Assessment, Liberty has zero reported PCPs, which reflects providers that list Liberty county on their licensure. This data does not account for physicians who have a primary office location in a neighboring county but who have satellite offices or otherwise provide services in Liberty county [14]. Missing data for other counties was not included in our linear model and was performed separately using a bivariate analysis. The counties that were excluded in this model were Calhoun, Dixie, Franklin, Glades, Jefferson, Lafayette, Liberty, Taylor, and Union due to missing or unavailable data for child mortality rates and the percentage of disconnected youth.

**Table 1:** All 67 Florida counties with their respective demographic and socioeconomic variables Note: Data is represented as percentages with no N, as reported by the County Health Rankings & Roadmaps database. MHC, mental health counselor; N/A, not applicable; PCP, primary care provider

County	% Poor or fair health	Teen birth rate (per 10,000)	% Uninsured	PCP rate	MHC provider rate	High school graduation rate	% Single-parent household	% Severe housing problem	Child mortality rate	% Children uninsured	% Disconnected youth	% Free or reduced lunch	% Black	% American Indian	% Hispanic	% White
Florida	18	23	16	72	151	81	38	21	53	7	10	58	16	0.5	26	54
Alachua	19	14	12	152	587	81	35	21	74	6	6	50	20	0.4	10	61
Baker	20	46	12	38	93	82	30	15	72	6	30	48	14	0.4	3	81
Bay	19	40	14	53	241	76	37	17	66	7	11	52	11	0.8	7	77
Bradford	21	49	13	33	44	80	42	16	111	6	33	55	19	0.5	4	74
Brevard	16	20	13	74	151	86	36	16	48	6	9	49	10	0.5	10	75
Broward	17	17	16	73	162	80	39	25	46	8	9	62	28	0.4	30	37
Calhoun	24	47	15	44	14	82	36	14	N/A	7	35	57	13	1.3	6	77
Charlotte	15	24	16	65	107	85	35	17	49	8	12	55	6	0.4	7	84
Citrus	19	32	15	53	60	79	40	15	73	7	13	67	3	0.4	6	88
Clay	16	20	11	53	85	87	29	15	44	6	9	44	11	0.5	10	73
Collier	16	22	23	73	96	87	36	20	43	12	10	63	7	0.5	28	63
Columbia	22	44	13	46	175	71	37	16	75	6	28	58	18	0.6	6	73
DeSoto	26	46	24	24	83	64	40	19	64	10	22	65	12	1.1	31	55
Dixie	26	44	15	8	6	92	44	13	N/A	7	34	78	10	0.5	4	84
Duval	18	31	13	85	178	79	42	19	78	6	12	51	29	0.4	10	53
Escambia	18	33	12	65	175	78	39	14	77	5	8	58	23	0.9	6	64
Flagler	15	21	15	51	53	80	37	17	39	8	11	58	10	0.4	10	75
Franklin	19	61	17	28	59	69	33	18	N/A	9	N/A	39	13	0.9	5	79
Gadsden	25	42	16	17	115	57	58	18	82	7	18	76	55	0.6	10	33
Gilchrist	21	38	17	36	41	93	30	13	91	9	35	55	5	0.7	6	87
Glades	22	24	26	7	N/A	85	38	15	N/A	15	N/A	48	13	5.4	21	60
Gulf	21	37	14	42	68	84	33	18	110	7	30	60	17	0.6	5	75
Hamilton	24	51	15	16	7	70	45	14	115	7	36	64	33	1	10	55
Hardee	28	55	21	13	16	69	40	20	39	9	18	64	7	1.2	44	47
Hendry	26	50	26	35	45	83	46	22	53	11	17	66	11	2.1	53	33
Hernando	19	23	15	54	76	84	36	16	50	7	12	65	5	0.5	13	78
Highlands	21	38	18	62	69	70	35	16	52	8	18	75	10	0.7	20	67
Hillsborough	19	26	14	83	170	81	40	20	59	6	9	59	16	0.5	28	49
Homes	23	50	16	36	82	74	39	15	76	8	25	52	7	1	3	87
Indian River	17	27	17	66	123	85	33	17	46	9	11	57	9	0.4	12	76
Jackson	25	39	14	41	117	72	44	13	72	7	18	65	26	0.8	5	65
Jefferson	18	30	14	21	38	56	40	15	N/A	8	27	80	34	0.4	4	60
Lafayette	22	32	21	12	12	88	22	16	N/A	12	47	59	14	0.8	13	71
Lake	16	29	15	71	82	77	37	17	59	7	13	63	10	0.6	15	70
Lee	17	26	19	63	100	78	40	19	53	9	10	54	8	0.5	21	68
Leon	15	12	11	84	207	89	39	22	54	6	6	41	31	0.3	6	57
Levy	22	33	18	19	17	80	42	15	79	8	17	63	9	0.7	8	80
Liberty	23	47	15	N/A	128	86	41	12	N/A	8	N/A	51	19	1.2	7	72
Madison	23	37	15	11	45	73	46	20	88	7	26	58	38	0.7	5	55
Manatee	16	32	16	56	97	81	38	18	49	7	11	55	8	0.5	16	71
Marion	20	35	16	58	90	79	41	16	74	6	14	66	12	0.5	13	71
Martin	14	19	17	67	163	86	31	18	54	10	8	45	5	0.9	14	78
Miami-Dade	22	18	20	80	151	80	41	31	46	7	11	70	16	0.3	68	13
Monroe	15	19	20	75	201	79	36	27	59	12	10	52	6	0.5	24	67
Nassau	16	27	12	46	111	91	32	15	52	7	10	49	6	0.5	4	87
Okaloosa	16	31	13	79	155	85	31	16	56	7	8	45	10	0.7	9	74
Okeechobee	25	52	23	49	53	70	42	18	70	10	20	82	8	1.5	25	63
Orange	19	22	15	85	214	82	38	23	57	7	8	62	20	0.6	31	41
Osceola	24	27	16	43	138	85	36	24	45	7	13	57	10	0.8	54	32
Palm Beach	17	18	17	79	189	84	37	23	44	8	9	59	18	0.6	22	55
Pasco	19	23	15	59	73	81	34	16	47	6	13	56	5	0.5	15	75
Pinellas	16	23	14	90	186	81	41	19	51	6	10	52	10	0.4	10	74
Polk	20	33	16	49	83	75	41	18	63	6	13	54	15	0.7	22	59
Putnam	25	50	17	42	57	66	49	20	80	7	18	66	16	0.7	10	71
St. Johns	12	11	10	94	114	90	21	16	39	5	7	23	5	0.3	7	83
St. Lucie	18	24	18	38	146	86	37	21	46	7	10	66	20	0.5	19	58
Santa Rose	16	25	12	72	55	85	27	14	43	5	11	44	6	0.9	6	83
Sarasota	13	19	16	78	168	84	34	17	43	9	8	51	4	0.3	9	83
Seminole	16	14	11	77	151	88	30	18	43	6	7	47	11	0.4	21	61
Sumter	14	47	12	36	39	83	36	12	73	7	20	60	7	0.4	6	85
Suwannee	24	42	17	15	42	85	40	16	75	8	30	62	13	0.7	9	76
Taylor	22	51	15	35	24	69	37	11	0	7	32	61	19	1	4	73
Union	23	49	12	24	72	80	38	16	0	6	33	55	22	0.5	6	69
Volusia	18	25	15	69	127	75	41	19	53	6	11	65	10	0.5	14	72
Wakulla	17	27	12	30	40	84	30	13	57	6	22	47	13	0.8	4	80
Walton	20	36	18	51	66	80	32	20	42	10	13	53	5	0.8	6	84
Washington	23	44	15	24	18	74	36	14	66	7	17	66	15	1.4	4	78

Almost all the parameter estimates used in our linear model were found to be significant factors in burn rate differences between counties. The PCP rate and percentage of American Indians were found to not have a significant difference in the pediatric burn rates for all counties, excluding those mentioned above. The results are outlined in Table 2 with parameter estimates, p-values, standard errors, and upper and lower confidence limits for all variables.

**Table 2:** Effect of each socioeconomic factor on pediatric burn rates across Florida PCP, primary care provider

Variable	Estimate	Standard error	p-value	Lower class limit	Upper class limit
% Poor or fair health	0.06	0.08	<0.0001	0.04	0.73
Teen birth rate	0.01	0.003	<0.05	0.002	0.01
% Uninsured	0.05	0.01	<0.0001	0.03	0.08
PCP rate	-0.0007	0.001	0.57	-0.003	0.002
Mental health provider rate	0.001	0.0003	<0.0001	0.0009	0.002
High school graduate rate	0.03	0.003	<0.0001	0.02	0.03
% Single-parent household	0.03	0.005	<0.0001	0.02	0.04
% Severe housing problems	-0.03	0.007	<0.05	-0.04	-0.01
Child mortality rate	0.004	0.001	<0.05	0.001	0.007
% Children uninsured	-0.09	0.02	<0.0001	-0.13	-0.06
% Disconnected youth	-0.02	0.004	<0.0001	-0.02	-0.008
% Free or reduced lunch	-0.01	0.002	<0.0001	-0.02	-0.009
% Black	0.05	0.01	<0.05	0.02	0.08
% American Indian	0.01	0.06	0.82	-0.11	0.14
% Hispanic	0.04	0.02	<0.05	0.01	0.06
% White non-Hispanic	0.05	0.01	<0.05	0.02	0.08

An increase in teen birth rate, percentage of uninsured, percentage of single-parent households, child mortality rate, and percentage of Blacks, Hispanics, and White non-Hispanics are all associated with significantly higher pediatric burn rates, some as high as two-fold increases (p < 0.05). While there was an increase in the percentage of severe housing problems, the percentage of uninsured children, the percentage of disconnected youth, and the percentage of children eligible for free or reduced lunch all showed a significant decrease in pediatric burn rates (p < 0.05). These changes in burn rate associated with a one-unit change of the variable predictor, represented by the parameter estimate, are detailed above.

**Figure 2:**
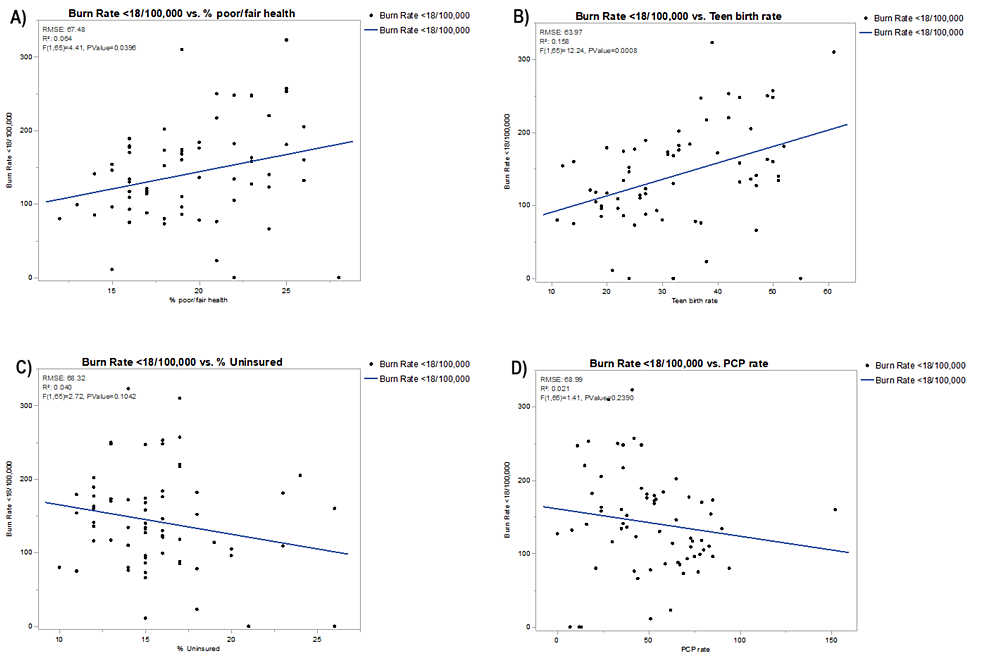
Bivariate analysis of pediatric burn rates against (A) poor or fair health, (B) teen birth rate, (C) % uninsured, and (D) PCP rate PCP, primary care provider; RMSE, root-mean-square error

**Figure 3:**
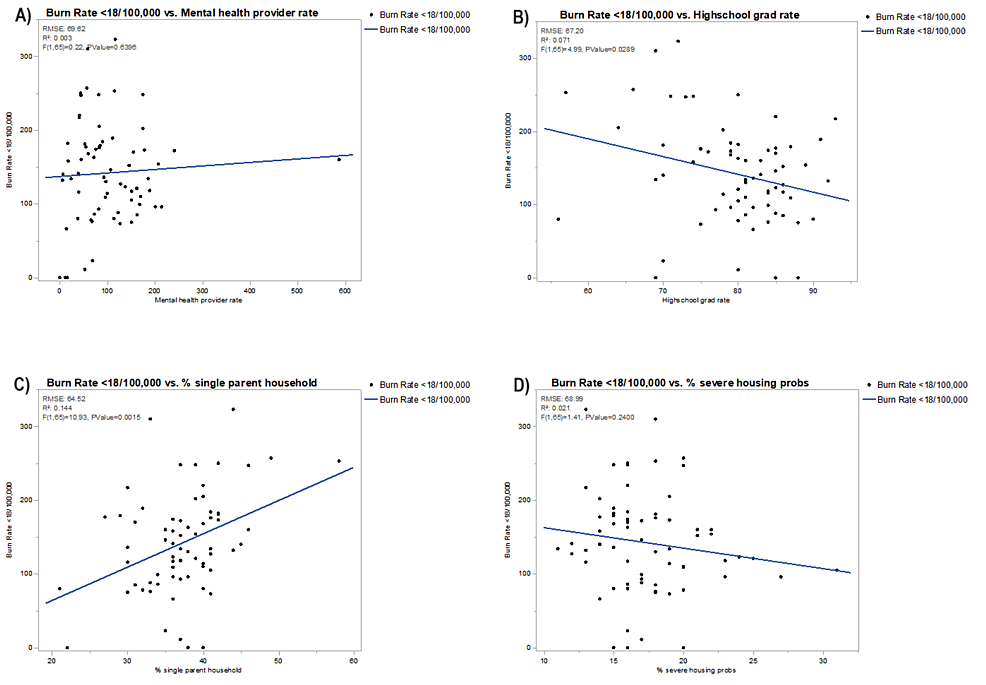
Bivariate analysis of pediatric burn rates against (A) mental health provider rate, (B) high school graduation rate, (C) % single-parent household, and (D) % severe housing problems RMSE, root-mean-square error

**Figure 4:**
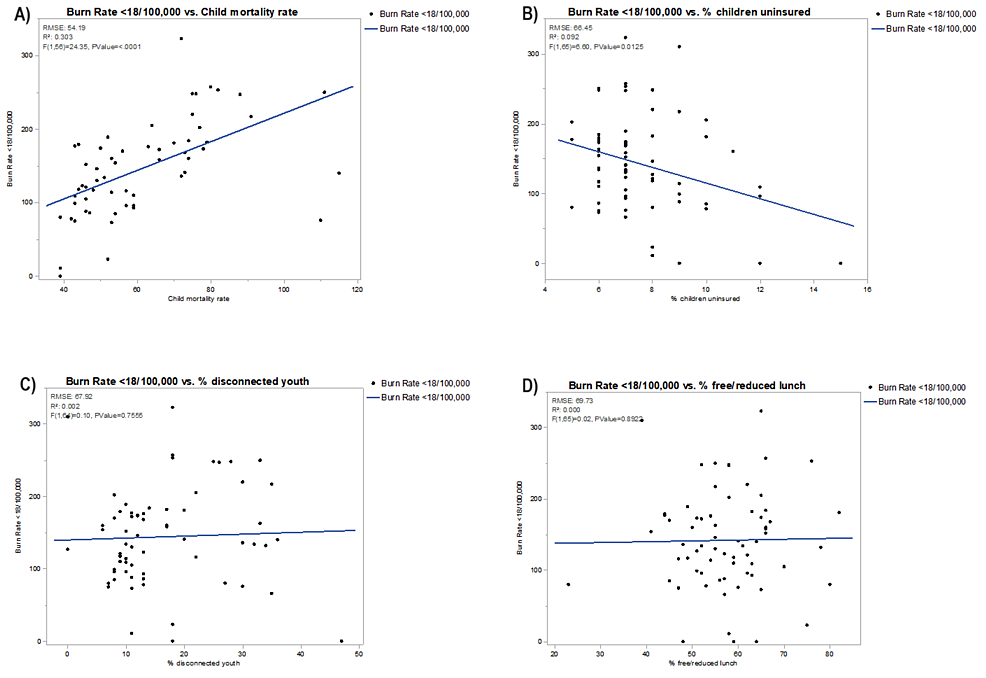
Bivariate analysis of pediatric burn rates against (A) child mortality rate, (B) % children uninsured, (C) % disconnected youth, and (D) % free or reduced lunch RMSE, root-mean-square error

**Figure 5:**
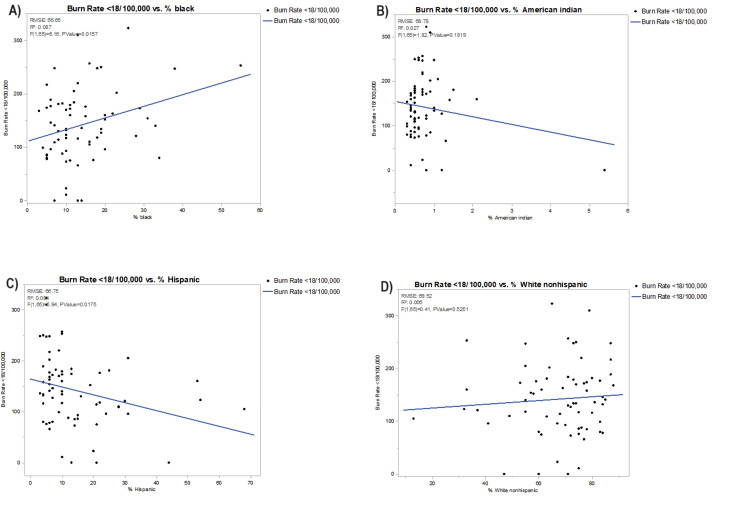
Bivariate analysis of pediatric burn rates against (A) % Black, (B) % American Indian, (C) % Hispanic, and (D) % White non-Hispanic RMSE, root-mean-square error

In Figures 2-5, each bivariate analysis of our independent variables crossed against our outcome variable of pediatric burn rate is represented. The R-squared value and root-mean-square error are included. Liberty county was included in the univariate analysis for pediatric burn rate versus PCP rate of zero for the aforementioned reasoning. Calhoun, Dixie, Franklin, Glades, Jefferson, Lafayette, Liberty, Taylor, and Union were excluded from the pediatric burn rate versus mortality rate due to unavailable data. Glades county was excluded from the analysis of the pediatric burn rate versus the percentage of disconnected youth due to unavailable data.

## Discussion

The research findings here suggest that lower sociodemographic counties in Florida have significantly higher burn rates compared to higher sociodemographic areas, but the interplay of each is a little more complicated than we hypothesized or previous literature has suggested thus far. Although we used various variables to represent the sociodemographic status of counties, most of them, excluding the PCP rate and percentage of American Indians, seemed to play a collective role in pediatric burn rates. Interestingly, our multivariate analysis revealed that an increase in high school graduation rates and mental health providers is associated with a decrease in pediatric burn rates, accounting for other variables as well. Furthermore, an increase in severe housing problems, the percentage of children uninsured, the percentage of disconnected youth, and the percentage of children receiving free or reduced lunch show a decrease in the pediatric burn rate. While these SDOH factors may be statistically significant, it is difficult to assign true clinical or practical significance, as pediatric burn risk appears to be a highly multifaceted issue.

Although multivariate analysis shows some connection between most of our variables and pediatric burn rate, univariate analysis has less significance for some of the variables tested. PCP rate and percentage of American Indians remained insignificant during univariate analysis, suggesting these variables play no role in pediatric burn rate when accounting for all other variables. However, our univariate analysis shows that the percentage of the total population uninsured, mental health care providers, the percentage of those with severe housing problems, the percentage of disconnected youth, the percentage of schoolchildren receiving free or reduced lunch, and the percentage of White non-Hispanics become nonsignificant, suggesting these variables alone do not account for pediatric burn rates. The use of a multivariate analysis here allowed for control of several independent variables that may have affected the outcome of pediatric burn rates and understanding what, if anything, may have significantly played a role. The additional univariate analysis utilized in this study allowed for subsequent individual variable analysis to illustrate a potential binary correlation between the SDOH variables and pediatric burn rates. This study shows both the individual SDOH factor effect on pediatric burn rates as well as the potential interplay and confounding effects within each.

Ethnicity and race

Children constitute a significant portion of burn victims in most studies from developing countries [15]. Although we did not categorize our pediatric patients by age, previous studies suggest that 11- to 16-year-olds account for most burn injuries; however, ages 0-5 are at the highest mortality risk from burn injuries [15]. Secondly, public education has been revealed as a factor in decreasing burn injuries in previous studies, which may also be evident from our study showing counties with higher graduation rates to be associated with lower pediatric burn rates [8,9,10,16]. Race and ethnicity have shown striking differences in burn rates in the US. Data for pediatric burn rates and race or ethnicity is sparse, but multiple studies have shown that total population burn rates are higher in African Americans and lowest in Asians [16-18]. Mierley and Baker performed a study on the demographics of fatal house fires in Baltimore over a three-year period, showing that the difference in burn rates between African Americans and Whites diminishes as income increases for both ethnicities [19]. The same studies showing increased burn rates for African Americans reported intermediate rates for Native Americans, White non-Hispanics, and Hispanics [16-18]. Our study of pediatric burn rates also concludes that African Americans have higher burn rates when accounting for race alone as well as other sociodemographic factors. Moreover, increasing Hispanic rates seem to have a lower pediatric burn rate association.

Housing burden

The home is full of hazards where children eat, sleep, play, and resolve conflicts. Most home environments are not configured to minimize the risk of injury to children. For example, areas of the kitchen where hot foods and liquids are being prepared are often a location for children to receive scald burns. Caretakers often find themselves involved in multiple tasks while preparing meals, including caring for younger children [15]. This can lead to children being unattended for a short time where they can reach these hot liquids and substances, leading to devastating burn injuries. The evidence in this study suggests that a major factor in pediatric burn injuries is the rate of single-parent households. As the percentage of single-parent households increases in counties, there is a significant increase in burn injuries among pediatric patients. Secondly, severe housing problems seem to have an inverse relationship with pediatric burn injuries. Severe housing problem data is represented by the percentage of households with one or more of the following problems: lack of complete kitchen facilities, lack of complete plumbing facilities, household overcrowding, and severe cost burden. This was found to be nonsignificant when comparing pediatric burn rates with severe housing problems alone, but becomes significant when accounting for all other variables, suggesting that these other variables also play a role in severe housing problems that lead to decreased pediatric burn rates. According to the WHO’s Global Burn Registry, a majority of cooking-related burn injuries occur in middle- and upper-income nations [20]. We hypothesize that the decrease in burn rates among the Florida population may further support the literature, potentially due to the lack of hazards such as kitchen facilities.

Insurance status

Previous literature on insurance rates and burns is limited. However, these studies reported that being uninsured was independently associated with an increased risk of mortality after burn injury and all-cause mortality, but Medicaid expansion was not associated with a decrease in mortality [21-23]. A study performed by Deghayli et al. on insurance coverage of pediatric burns in Switzerland versus the US revealed differences in burn rates between Switzerland, which has a universal healthcare system, and the US, which does not [24]. Private and commercial insurance groups have seen more burn cases in both pediatric and adult patients compared to government insurance bodies. Additionally, Switzerland’s governmental insurance automatically covers all burn-related procedures, while the American system often requires pre-authorization [24]. Our study found interesting results on insurance coverage and pediatric burn rates. First, the percentage of the total population insured, including adults, seems to be a nonsignificant factor in pediatric burn rates in our univariate analysis; however, when accounting for all other variables, an increase in the population uninsured has a decreased rate of pediatric burn injuries. Secondly, an increase in children who are uninsured seems to have a significant decrease in the outcome of pediatric burn injuries during both univariate and multivariate analyses. We believe that those who are uninsured will less often seek care for injuries such as burns. The degree of burn (TBSA and depth) was not extrapolated from this study, so it is difficult to determine whether insurance status would play less of a role in increased burn significance.

Mortality rate and burns

To our knowledge, this is the first study examining county mortality rates as an indicator of nonfatal burn rates. There is a plethora of literature comparing mortality rates from burn injuries in various demographics, but nothing to suggest that areas with an increased mortality rate also have increased pediatric burn rates. Snelling et al. published a study in 2021 reviewing burns and socioeconomic deprivation [25]. A 16-year retrospective review was performed of all acute adult burns assessed by Queen Elizabeth Hospital in Birmingham, United Kingdom. They concluded that the inequality in SES reflected a 4.5-time increase in males and a 3.9-time increase in females for avoidable or “amendable” deaths [25,26]. It seems obvious that lower SES areas have increased mortality rates, which is also reflected in our findings that a lower SES area, represented by increased mortality rates, has higher pediatric burn rates.

Limitations and strengths

It is important to consider some limitations of this study. First, data published by Florida Health CHARTS that is separated by mechanism uses categorical data for ages [27]. If an age group has less than five burn injuries, it is reported as “<5”. This analysis used 0 as a marker to not overestimate burn rates for counties, but it does limit the possible inclusion of age groups that may have lower burn rates. Secondly, Florida is a large state with the third-largest population, behind California and Texas. The large population makes public health research strong, but unfortunately, there is a vast distribution of population, with some cities making up a large majority of the Florida population. This would lead to an underrepresentation of some areas. Lastly, many of the linear regression models had a relatively low R-squared value, ranging from 0.001 to 0.30. Although many of our regression models show a significant association (p < 0.05), there seems to be a somewhat large proportion of variance for the dependent variables, limiting the generalizability and strength of the findings. In this context, these results further underscore the need for further research to explore and understand additional factors that may contribute to pediatric burn rates and ultimately enhance the predictive power of the models. Inherent strengths of this study include the use of univariate and multivariate analyses, which allowed for control of potentially confounding variables. Additionally, this study analyzed a wide range of potential SDOH factors in order to generate a comprehensive understanding of pediatric burn rates. Furthermore, to our knowledge, this is the first study examining multiple possible sociodemographic factors leading to increased pediatric burn rates in the US.

## Conclusions

This study represents increased pediatric burn rates for variables representing the sociodemographic status of all 67 Florida counties. Children in disadvantaged populations may be at increased risk of burn incidence and significant injury, and here we aim to highlight this potential disparity. This study adds to the limited body of literature surrounding the interplay of sociodemographic factors and burn rates in children, especially in the Florida community. While the study has its limitations, it provides additional insight into this topic and highlights the need for continued research, public health outreach, increases in education, and improvements in home environments as areas of focus to reduce these risks, especially for those in disadvantaged populations. Burn injuries are preventable trauma that leads to major healthcare costs as well as both physical and mental disability in patients. Research needs to continually aim to better identify the underlying associated variables and meet the needs of lower sociodemographic areas to improve burn rates in vulnerable populations, such as children, who are at increased risk of injury.
